# Where do adults see alcohol marketing? Insight from a cross-sectional survey in the United Kingdom

**DOI:** 10.1093/pubmed/fdaf118

**Published:** 2025-09-26

**Authors:** Nathan Critchlow, Anne Marie MacKintosh, Allison Ford

**Affiliations:** Institute for Social Marketing and Health, Faculty of Health Sciences and Sport, University of Stirling, Stirling, FK9 4LA, Scotland (United Kingdom); Institute for Social Marketing and Health, Faculty of Health Sciences and Sport, University of Stirling, Stirling, FK9 4LA, Scotland (United Kingdom); Institute for Social Marketing and Health, Faculty of Health Sciences and Sport, University of Stirling, Stirling, FK9 4LA, Scotland (United Kingdom)

**Keywords:** adults, alcohol advertising, alcohol marketing, alcohol pricing, alcohol promotion, alcohol use, surveys

## Abstract

**Background:**

There is limited insight about the reach of alcohol marketing among adults in the United Kingdom (UK). We therefore examined awareness across a range of marketing activities and sources and how this differed by degree of alcohol use.

**Method:**

An online cross-sectional survey with a nonprobability adult sample (18+) in the UK (*n* = 6021). Participants self-reported past-month awareness of alcohol marketing and special price deals from companies/brands, off-trade shops (e.g. supermarkets), online retailers, and on-trade venues (e.g. pubs/bars). Alcohol use was assessed using the AUDIT-C (coded: nondrinkers, lower-risk drinkers, higher-risk drinkers, not stated).

**Results:**

For alcohol companies/brands, 65.9% had seen advertising (e.g. on TV) and 79.5% had seen wider marketing (e.g. sponsorship). For off-trade shops, 86.8% had seen marketing (e.g. product displays) and 76.6% had seen special price deals (e.g. multi-buy discounts). For online retailers, 30.8% had seen marketing (e.g. leaflets/flyers) and 52.4% had seen special price offers. For on-trade venues, 69.0% had seen marketing (e.g. posters/leaflets/flyers) and 52.1% had seen special price offers. The odds of reporting awareness were generally lower among nondrinkers and higher among higher-risk drinkers (vs. lower-risk).

**Conclusion:**

Adults see alcohol marketing through various activities and sources, with awareness generally increasing with degree of alcohol use.

## Introduction

Alcohol consumption is a leading risk factor for many health and social harms, which, in turn, generate a substantial societal burden in the United Kingdom (UK).^1^ Multiple systematic reviews have concluded that alcohol marketing is associated with consumption.^2^ Alcohol marketing refers to all the activities used by producers and places that sell alcohol (e.g. shops, pubs, and bars) to increase the visibility and salience of alcohol products and brands. In the UK, this includes, but is not limited to, advertising (e.g. TV^3^, posters,^4^^,^^5^ radio^6^ or magazines^7–9^), sponsorship,^10^^,^^11^ packaging and glassware,^12^^,^^13^ price offers,^14^^,^^15^ displays in shops,^16^ and online marketing.^17–20^ This array of activities is collectively referred to as the marketing ‘Ps’ (product, place, price, and promotion) or ‘marketing mix’.^21^^,^^22^

To limit the role that marketing plays in driving consumption, several European countries have introduced statutory restrictions on how alcohol can be marketed.^23^^,^^24^ In the UK, alcohol marketing remains mostly self-regulated by the alcohol and marketing industries.^25–27^ Potential restrictions have, however, received regional attention. The Scottish Government consulted on whether to implement restrictions on alcohol advertising and promotions in 2022.^28^ Some local authorities in England have already introduced their own restrictions on alcohol advertising.^29^^,^^30^ The UK Government, however, has previously implemented population-level marketing restrictions, such as longstanding controls on tobacco products^31–33^ and recent restrictions for food and drinks high in fat, salt, and/or sugar.^34^

Understanding where and how consumers are exposed to alcohol marketing is key to informing policy development and decisions about how marketing should be controlled. In the UK, most research examining the reach of alcohol marketing focuses on young people.^35–41^ To date, we have not found a UK study that has quantified adult awareness across a range of alcohol marketing activities. Instead, adult research is limited to qualitative studies focused on specific subpopulations or aspects of marketing,^13^^,^^42–46^ studies that use proxy measurements for marketing exposure (e.g. TV viewership data),^47–49^ experimental studies that manipulate exposure,^50^^,^^51^ or studies examining public opinion on marketing restrictions.^52–54^

Addressing the absence of research about the reach of alcohol marketing among adults in the UK is important, given that they are legitimate targets for marketing and the primary source of most alcohol sales.^55^^,^^56^ Some groups in the adult population may also have heightened susceptibility to marketing, such as those looking to cut down or avoid alcohol.^57^^,^^58^ We therefore examine awareness of a wide range of alcohol marketing activities among adults in the UK and explore to what extent, if at all, awareness varies by degree of alcohol use and risk. We further build on existing quantitative research in the UK by also examining, in depth, marketing and price deals from places that sell alcohol (e.g. shops, pubs, or online retailers).

## Methods

### Design

A self-report online cross-sectional survey with adults (18+ years) in the UK, conducted 8–26 November 2024. The study was approved by the University of Stirling’s General University Ethics Panel (GUEP 2024 19707 14487).

### Survey development

Survey design, including the marketing activities and sources included, was informed by several activities. First, we conducted six public involvement groups, each with five participants, to explore their experiences of alcohol marketing, examine the language they used, and gauge the suitability of measurement approaches. The groups contained either male or female participants, and each group focused on different age bands (from 18–24 years to 55+ years). We conducted five groups with current drinkers and one with nondrinkers. The groups were held in-person near Glasgow (Scotland), in a local community venue, and lasted ~ 90 minutes. The discussions were led by two experienced researchers (AM and AF) and used visual stimuli, such as images of alcohol brands and marketing, to facilitate discussion. Participants were recruited using a local market recruiter and received a voucher and reimbursement of travel expenses.

Following survey drafting, we conducted 16 one-to-one cognitive interviews with members of the public to test their comprehension of, and ability to respond to, draft questions. Initially, we conducted four interviews with current drinkers, covering varied ages and sexes, to test early question concepts. After incorporating feedback, we conducted 12 further interviews to test and refine questions, covering a mixture of ages, sexes, and drinking statuses. The interviews took place in person near Glasgow and lasted ~60 minutes. Participants were recruited by a local market recruiter and received a voucher and travel expenses.

The final draft survey instrument was reviewed by YouGov to check length and consistency, and identify opportunities to enhance participant experience (e.g. visually engaging response formats). Once scripted, the survey was tested by the researchers to address any issues with routing and logic. The final survey was piloted with ~100 participants, allowing us to ensure that the design performed as intended. The final survey took ~15 minutes to complete.

### Main survey recruitment

Main survey fieldwork was conducted by YouGov, a leading research data and analytics group,^59^ who recruited a sample broadly representative of the UK adult population (18+) from their nonprobability online panel. The panel has over 400 000 active panel members in the UK, recruited through a mixture of organic growth and targeted advertising and recruitment. Potential participants received a generic invitation to complete a survey via an e-mail or app notification. Those routed to this survey were then shown a study-specific landing page and consent form. Participants received YouGov points for completing the survey, which are redeemable for vouchers or monetary rewards once thresholds are reached.

Overall, 6900 panellists opened our survey landing page. Of these, 279 dropped out without indicating whether they consented to participate. Of the remaining 6621 panellists, 227 declined to participate and 236 dropped out across the survey. Of the 6158 participants registered as completing the survey, the 111 panellists who took part in the pilot were removed, as a small number of modifications were made prior to the main fieldwork. A further 26 panellists were excluded by YouGov in postfieldwork quality checks, e.g. due to missing data required for the weighting variable. This resulted in a final sample of 6021, a completion rate of 87% from the panellists routed to the landing page.

### Measures

#### Demography

YouGov provided data on age, sex, UK nation lived in, and social grade, with the latter categorized as ABC1 (‘higher’) or C2DE (‘lower’) according to the National Readership Survey categories (Table 1).^60^

#### Degree of alcohol use and risk

All participants completed the first question from the three-item Alcohol Use Disorders Identification Test–Consumption (AUDIT-C),^61^ which measures frequency of drinking. Those who indicated any alcohol use (‘current drinkers’) completed the remaining AUDIT-C items about units drunk on a typical occasion (one unit = 10 ml/8 g of pure alcohol) and frequency of heavy episodic drinking (≥8 units if male/≥6 if female on a single occasion). Participants were provided with an infographic detailing the typical number of units in various alcoholic drinks to aid response. For analysis, we categorized participants as either: nondrinkers (‘never’ on first AUDIT-C item); lower-risk drinkers (scored 1–4 on AUDIT-C); higher-risk drinkers (scored ≥ 5 on AUDIT-C); drinking status not specified (‘don’t know’/‘prefer not to say’ to first AUDIT-C item); or risk not specified (‘don’t know’/‘prefer not to say’ to second and/or third AUDIT-C items).

#### Awareness of alcohol marketing

Participants were asked to report past month awareness across 26 marketing activities from both alcohol companies and brands (Table 2) and places that sell alcohol (Table 3), the latter of which included questions on physical off-trade retailers (‘shops’, e.g. supermarkets), online retailers (e.g. supermarket websites, subscription services, takeaway/grocery delivery apps), and on-trade premises (‘venues’; e.g. pubs, bars, nightclubs). For each activity, participants were coded into those who reported past month awareness (‘yes’) versus all nonaffirmative categories (i.e. ‘no’ and ‘don’t know’). A past month recall period is consistent with previous research on alcohol marketing^38^^,^^62^ and was considered an appropriate and feasible reference period in the development activities. The wording of activities has been shortened for brevity in the results. The full wording as presented to participants is reported in Supplementary File 1.

#### Awareness of special price deals

For places that sell alcohol, participants were also asked to report what special price deals they had seen for alcohol in the past month. Awareness was captured on four special price deals for shops, five for online retailers, and five for venues (Table 4). For each source, participants were asked to select all that apply or indicate ‘none of the above’ or ‘don’t know’. Special price deals were measured in granular detail, as opposed to a single generic item, to capture the range of promotional activity highlighted in the development stages. The wording of price offers has been shortened for brevity in the results. The full wording as presented to participants is reported in Supplementary File 1.

### Analysis

Data were analysed using SPSS version 29.^63^ Descriptive data are adjusted using sample weights provided by YouGov based on sex, age, region, social grade, and ethnicity. Binary logistic regression models were computed to examine whether awareness of each marketing activity or special price deal was associated with degree of alcohol use. In each model, reporting any past-month awareness was the dependent variable (yes vs. the combined nonaffirmative categories) and degree of alcohol use was the key independent variable. For degree of alcohol use, lower-risk drinkers formed the reference category versus nondrinkers or higher-risk drinkers. We included ‘drinking status not specified’ and ‘drinking risk not specified’ as separate categories to ensure that the models utilised the full sample, but the parameters for these are not reported due to limited space and because their comparison to the reference category is not meaningful. Each model controlled for age, sex, UK nation, and social grade. The model outcomes are expressed via adjusted odds ratios (*OR_Adj_*), with 95% confidence intervals (95% CI), and *P-*values (with *P* = .05 the accepted threshold for statistical significance).

## Results

### Sample characteristics and alcohol use

Table 1 reports the sample demographics and degree of alcohol use and risk. A fifth of participants (19.8%) were nondrinkers, two-fifths (40.3%) were lower-risk drinkers, and a third (34.7%) were higher-risk drinkers. The remainder were not specified for drinking risk (3.4%) or drinking status (1.8%).

**Table 1:** Sample characteristics and degree of alcohol use and risk.

	Unweighted		Weighted	
Variable	%	*n*	%	*n*
**Sex**				
Male	48.7	2932	48.5	2921
Female	51.3	3089	51.5	3100
**Age band**				
18–24 years	10.3	618	10.4	625
25–39 years	25.2	1517	25.4	1528
40–54 years	24.3	1463	24.3	1462
55–64 years	16.5	992	16.3	981
65–74 years	17.2	1038	17.2	1034
75+ years	6.5	393	6.5	392
**UK nation**				
England	84.0	5060	84.0	5058
Scotland	7.9	478	8.0	482
Wales	5.0	304	5.0	301
Northern Ireland	3.0	179	3.0	181
**Social grade**				
C2DE (lower)	45.6	2743	45.4	2732
ABC1 (higher)	54.4	3278	54.6	3289
**Alcohol use and risk**				
Nondrinker	19.8	1192	19.8	1192
Lower-risk drinker (1–4 AUDIT-C)	40.3	2425	40.3	2427
Higher-risk drinker (≥5 AUDIT-C)	34.7	2090	34.7	2089
Drinking risk not specified	3.4	206	3.4	206
Drinking status not specified	1.8	108	1.8	108

Notes: Base = all participants (*n* = 6021); drinking risk not specified = participants who reported ‘don’t know’ or ‘prefer not to say’ to the second (alcohol units drunk on a typical occasion) and/or third (frequency of heavy episodic drinking) items on the AUDIT-C.

### Advertising from alcohol companies and brands

Two-thirds of participants (65.9%) recalled seeing at least one alcohol advertising activity from companies and brands in the past month, with awareness ranging from 37.2% for posters and billboards to 5.5% for cinema adverts (Table 2).

**Table 2:** Past month awareness of advertising and wider marketing activities from alcohol companies and brands and the associations with degree of alcohol use and risk among adults in the UK.

	Aware	Nondrinkers *(vs. lower-risk drinkers)*			Higher-risk drinkers *(vs. lower-risk drinkers)*		
	*%*	*OR* _*Adj.*_	*95% CI*	*P*	*OR* _*Adj.*_	*95% CI*	*P*
**Advertising from companies and brands**							
Posters or billboards (e.g. in the street or on public transport)	37.2	0.75	0.64, 0.88	<.001	1.09	0.96, 1.23	.209
Broadcast TV (incl. programme sponsorship)	30.1	0.73	0.62, 0.86	<.001	1.15	1.01, 1.30	.037
Catch-up/streaming TV services	26.7	0.78	0.66, 0.92	.003	1.13	0.99, 1.30	.063
Popping up on online (e.g. browsing websites or using apps)	26.1	0.91	0.77, 1.08	.298	1.16	1.01, 1.33	.039
Newspapers or magazines	19.9	0.79	0.65, 0.96	.015	1.17	1.01, 1.35	.036
Podcasts/audio streaming (incl. show/episode sponsorship)	10.5	0.81	0.63, 1.03	.087	1.06	0.87, 1.29	.578
Radio	7.4	0.62	0.46, 0.83	.002	0.94	0.76, 1.18	.613
Cinema	5.5	0.67	0.47, 0.97	.034	1.45	1.13, 1.88	.004
**Wider marketing from companies and brands**							
Brand names or logos on packaging	63.8	0.47	0.40, 0.54	<.001	1.60	1.40, 1.83	<.001
Sports sponsorship (e.g. teams, events, tournaments)	30.6	0.94	0.80, 1.11	.484	1.30	1.14, 1.48	<.001
Celebrity endorsement	29.5	0.95	0.81, 1.12	.541	1.13	0.99, 1.29	.076
Cultural event sponsorship (e.g. concerts, festivals)	25.1	0.85	0.71, 1.02	.077	1.43	1.24, 1.65	<.001
Competitions or giveaways linked to an alcohol company	21.3	0.76	0.63, 0.91	.003	1.19	1.03, 1.38	.019
Social media posts by alcohol companies or brands	19.8	0.62	0.51, 0.76	<.001	1.42	1.22, 1.64	<.001
Free trials, tasters, or samples	15.4	0.77	0.63, 0.95	.016	1.22	1.03, 1.43	.019

Notes: Base = All participants (*n* = 6021); descriptive frequency (%) data are weighted; Logistic regression models are unweighted, but control for age band, sex, UK nation, and social grade; data for drinking status not specified (*n* = 108; unweighted) and drinking risk not specified (*n* = 206; unweighted) included in each model but not reported in the table; see Supplementary File 1 for full wording of activities as presented to participants in the survey.

For 6/8 advertising activities, the odds of reporting awareness were lower among nondrinkers versus lower-risk drinkers (range *OR_Adj_*:0.62 to 0.79; *P* < .001 to .034) (Table 2). The exceptions, in which awareness did not differ between nondrinkers and lower-risk drinkers, were adverts popping up online (*P* = .298) and adverts on podcasts or audio streaming services (incl. show/episode sponsorship) (*P* = .087).

For 4/8 advertising activities, the odds of reporting awareness were higher among higher-risk drinkers versus lower-risk drinkers (range *OR_Adj_*:1.15 to 1.45; *P* = .004 to .039) (Table 2). The exceptions, in which awareness did not vary by drinking risk, were adverts on the radio (*P* = .613), podcasts/audio streaming services (*P* = .578), posters or billboards (*P* = .209), and catch-up/streaming TV services (*P* = .063).

### Wider marketing from alcohol companies and brands

Four-fifths of participants (79.5%) recalled seeing at least one wider marketing activity from companies or brands in the past month, with awareness ranging from 63.8% for names/logos on packaging to 15.4% for trials, tasters, or samples (Table 2).

For 4/7 wider marketing activities, the odds of reporting awareness were lower among nondrinkers versus lower-risk drinkers (range *OR_Adj_*:0.47 to 0.77; *P* < .001 to .016) (Table 2). The exceptions, in which awareness did not differ between nondrinkers and lower-risk drinkers, were sports sponsorship (*P* = .484), celebrity endorsement (*P* = .541), and cultural event sponsorship (*P* = .077).

For 6/7 wider marketing activities, the odds of reporting awareness were higher among higher-risk drinkers versus lower-risk drinkers (range *OR_Adj_*:1.19 to 1.60; *P* < .001 to .019) (Table 2). The only exception was celebrity endorsement, in which awareness did not differ by drinking risk (*P* = .076).

### Alcohol marketing and price deals from shops

Most participants (86.8%) recalled seeing at least one marketing activity from shops in the past month, with awareness ranging from 78.7% for displays of alcohol (e.g. at the end of aisles) to 19.6% for social media posts about alcohol from shops (Table 3).

**Table 3:** Past month awareness of marketing from off-trade retailers (shops), online retailers, and on-trade venues (e.g. pubs, bars, nightclubs) and the associations with degree of alcohol use and risk among adults in the UK.

	Aware	Nondrinkers *(vs. lower-risk drinkers)*			Higher-risk drinkers *(vs. lower-risk drinkers)*		
	*%*	*OR* _*Adj.*_	*95% CI*	*P*	*OR* _*Adj.*_	*95% CI*	*P*
**Marketing from off-trade retailers (shops)**							
Display in shops (e.g. end of aisles, at checkouts)	78.7	0.53	0.45, 0.62	<.001	1.44	1.23, 1.70	<.001
Signs or posters (e.g. on shop windows, doors, or shelves)	55.8	0.75	0.66, 0.87	<.001	1.31	1.16, 1.48	<.001
Store leaflets, flyers, or magazines that show alcohol	50.3	0.77	0.67, 0.89	<.001	1.04	0.93, 1.17	.496
Social media posts about alcohol from shops	19.6	0.63	0.52, 0.76	<.001	1.10	0.95, 1.27	.194
**Marketing from online retailers**							
E-mail or app notifications from supermarkets or online retailers	16.7	0.63	0.51, 0.79	<.001	1.56	1.34, 1.82	<.001
Leaflets or flyers about an online retailer/subscription service	14.6	0.83	0.67, 1.03	.094	1.26	1.07, 1.49	.006
Social media posts by specialist retailers/subscription services	10.7	0.59	0.45, 0.78	<.001	1.60	1.33, 1.91	<.001
**Marketing from on-trade venues (e.g. pub, bars, clubs)**							
Brand names or logos *inside* a venue (e.g. drinks mats)	60.7	0.36	0.31, 0.42	<.001	2.44	2.13, 2.80	<.001
Brand names or logos *outside* a venue (e.g. signage)	39.6	0.68	0.58, 0.80	<.001	1.55	1.37, 1.76	<.001
Posters, flyers, or leaflets that promote a venue	27.2	0.85	0.72, 1.01	.064	1.36	1.19, 1.56	<.001
Social media posts about alcohol from venues	15.7	0.57	0.45, 0.71	<.001	1.51	1.29, 1.78	<.001

Notes: Base = All participants (*n* = 6021); descriptive frequency (%) data are weighted; Logistic regression models are unweighted, but control for age band, sex, UK nation, and social grade; data for drinking status not specified (*n* = 108; unweighted) and drinking risk not specified (*n* = 206; unweighted) included in each model but not reported in the table; see Supplementary File 1 for full wording of activities as presented to participants in the survey.

Compared to lower-risk drinkers, the odds of reporting awareness were lower among nondrinkers for all shop marketing activities (range *OR_Adj_*:0.53 to 0.77; all *P* < .001) and higher among higher-risk drinkers for displays in shops (*OR*_*Adj*._ = 1.44, *P* < .001) and signs or posters in shops (*OR*_*Adj*._ = 1.31, *P* < .001) (Table 3). Awareness of store leaflets, flyers, or magazines (*P* = 0.496) and social media posts about alcohol from shops (*P* = .194) did not vary by degree of drinking risk.

Three-quarters of participants (76.6%) had seen at least one special price deal for alcohol in shops in the past month, with awareness ranging from 49.6% for discounts linked to reward/membership schemes to 33.7% for price reductions (Table 4). Compared to lower-risk drinkers, the odds of reporting awareness of all special price deals from shops were lower among nondrinkers (range *OR_Adj_*:0.53 to 0.64; all *P* < .001) and higher among higher-risk drinkers (range *OR_Adj_*:1.25 to 1.86; all *P* < .001).

**Table 4:** Past month awareness of special price deals from off-trade retailers (shops), online retailers, and on-trade venues (e.g. pubs, bars, nightclubs) and the associations with degree of alcohol use and risk among adults in the UK.

	Aware	Non-drinkers *(vs. lower-risk drinkers)*			Higher-risk drinkers *(vs. lower-risk drinkers)*		
	*%*	*OR* _*Adj.*_	*95% CI*	*p*	*OR* _*Adj.*_	*95% CI*	*p*
**Price deals from off-trade retailers (shops)**							
Discounts linked to shop reward/membership schemes	49.6	0.54	0.47, 0.62	<0.001	1.69	1.49, 1.90	<0.001
Multi-buy/bulk discounts (e.g. 'three bottles of beer for £5′)	49.2	0.53	0.45, 0.61	<0.001	1.86	1.64, 2.11	<0.001
'Dine-in' deals including alcohol (e.g. 'dinner for two for £10′)	38.1	0.64	0.55, 0.74	<0.001	1.26	1.12, 1.42	<0.001
Price reductions (e.g. '30% off' or ‘price-matching’)	33.7	0.57	0.48, 0.67	<0.001	1.25	1.11, 1.42	<0.001
**Price deals from online retailers**							
Multi-buy/bulk discounts (e.g. ‘6 for £10 or 12 for £15’)	29.9	0.59	0.50, 0.70	<0.001	1.50	1.32, 1.70	<0.001
Price reductions (e.g. 'save 10%' or 'was £13.50, now £11′)	28.2	0.62	0.53, 0.74	<0.001	1.31	1.15, 1.49	<0.001
Introductory subscription offers (e.g. ‘first box half price’)	18.8	0.70	0.57, 0.86	<0.001	1.53	1.32, 1.77	<0.001
Discounts for regular deliveries (e.g. 'subscribe and save')	15.7	0.68	0.55, 0.85	<0.001	1.32	1.13, 1.54	<0.001
Discount codes (e.g. 'enter Oct24 at checkout for £10 off')	12.9	0.69	0.54, 0.88	0.002	1.53	1.29, 1.82	<0.001
**Price deals from on-trade venues (e.g. pub, bars, clubs)**							
Food and drink combination deals (e.g. ‘beer and burger’)	36.5	0.54	0.46, 0.63	<0.001	1.47	1.30, 1.66	<0.001
Multi-buy/bulk discounts (e.g. ‘two-for-one drinks’)	23.6	0.57	0.47, 0.69	<0.001	1.63	1.42, 1.86	<0.001
Temporary price reductions (e.g. ‘early bird’ offers)	22.2	0.53	0.44, 0.65	<0.001	1.49	1.30, 1.71	<0.001
Package deals that include alcohol	11.4	0.62	0.48, 0.80	<0.001	1.39	1.15, 1.68	<0.001
Discount vouchers/offers for venues that include alcohol	9.0	0.59	0.45, 0.78	<0.001	1.15	0.93, 1.40	0.193

Notes: Base = All participants (*n* = 6021); Descriptive frequency (%) data are weighted; Logistic regression models are unweighted, but control for age band, sex, UK nation, and social grade; Data for drinking status not specified (*n* = 108; unweighted) and drinking risk not specified (*n* = 206; unweighted) included in each model but not reported in the table; see Supplementary File 1 for full wording of special price deals as presented to participants in the survey.

### Alcohol marketing and price deals from online retailers

Almost a third of participants (30.8%) recalled seeing at least one alcohol marketing activity from online retailers in the past month, with awareness ranging from 16.7% for e-mail or app notifications from supermarkets/online retailers to 10.7% for social media posts by specialist alcohol retailers or subscription services (Table 3).

Compared to lower-risk drinkers, nondrinkers had lower odds of reporting awareness of e-mail or app notifications from supermarkets/online retailers (*OR_Adj_* = 0.63, *P* < .001) and social media posts by specialist alcohol retailers or subscription services (*OR_Adj_* = 0.59, *P* < .001) (Table 3). Awareness of leaflets or flyers about online alcohol retailers or subscription services did not differ between lower-risk drinkers and nondrinkers (*P* = .094). Compared to lower-risk drinkers, the odds of reporting awareness were higher among higher-risk drinkers for all online retailer marketing activities (range *OR_Adj_*:1.26 to 1.60; range *P* < .001 to .006).

Half of participants (52.4%) recalled seeing at least one special price deal for alcohol from online retailers in the past month, with awareness ranging from 29.9% for multi-buy/bulk discounts to 12.9% for discount codes (Table 4). Compared to lower-risk drinkers, the odds of reporting awareness of all special price deals from online retailers were lower among nondrinkers (range *OR_Adj_*:0.59 to 0.70; range *P* < .001 to .002) and higher among higher-risk drinkers (range *OR_Adj_*:1.31 to 1.53; all *P* < .001).

### Alcohol marketing and price deals from venues

Approximately two-thirds of participants (69.0%) recalled seeing at least one alcohol marketing activity from on-trade venues in the past month, with awareness ranging from 60.7% for brand names/logos inside a venue (e.g. on beer taps) to 15.7% for social media posts about alcohol from venues.

For 3/4 marketing activities from venues, the odds of reporting awareness were lower among nondrinkers compared to lower-risk drinkers (range *OR_Adj_*:0.36 to 0.68; all *P* < .001). The exception was posters, flyers, or leaflets that promoted a venue, in which awareness did not differ between nondrinkers and lower-risk drinkers (*P* = .064). For all marketing activities from venues, the odds of reporting awareness were higher among higher-risk drinkers compared to lower-risk drinkers (range *OR_Adj_*:1.36 to 2.44; all *P* < .001).

Half of participants (52.1%) recalled seeing at least one special price deal for alcohol from venues in the past month, with awareness ranging from 36.5% for food and drink combination deals to 9.0% for discount vouchers and offers (Table 4). Compared to lower-risk drinkers, the odds of reporting awareness of all special price deals from venues were lower among nondrinkers (range *OR_Adj_*:0.53 to 0.62; all *P* < .001). The odds were higher among higher-risk drinkers for most price deals (range *OR_Adj_*:1.39 to 1.63; all *P* < .001), except discount vouchers and offers (*P* = .193).

## Discussion

### Main findings of this study

Adults in the UK report seeing a range of alcohol marketing activities in the past month. This includes advertising and marketing from alcohol companies and brands, as well as a range of marketing and price deals from places that sell alcohol, such as shops, online retailers, and venues. Compared to lower-risk drinkers, awareness of marketing and price deals was generally lower among nondrinkers and higher among those drinking at higher risk.

### What is already known on this topic

Multiple reviews have concluded that marketing is associated with alcohol consumption, including among young people and vulnerable adult groups.^2^^,^^58^ There is a range of studies documenting the variety of activities that contribute to the alcohol ‘marketing mix' in the UK.^3–20^ While some studies have examined the reach of these activities among consumers, these only focus on young people and mostly focus on marketing from companies and brands.^35–41^ Existing research with adults in the UK is limited to qualitative studies on specific subpopulations or aspects of marketing,^13^^,^^42–46^ studies that use proxy measurements for marketing exposure,^47–49^ experimental studies that manipulate exposure,^50^^,^^51^ or studies assessing public views on marketing restrictions.^52–54^

### What this study adds

To our knowledge, this is the first study to quantitatively examine awareness of alcohol marketing among a diverse adult sample in the UK. A key strength is the iterative survey development stage. The public involvement groups were particularly informative in identifying and refining the breadth of marketing activities and sources to be measured. Through this comprehensive assessment, our analysis also builds on existing quantitative research, both in the UK and internationally, by demonstrating the importance of considering marketing activities and price deals from places that sell alcohol when mapping and measuring exposure. Together, these findings provide key insight to inform policy development around alcohol marketing in the UK.

As far as we are aware, this is also the first quantitative study in the UK to show that awareness of alcohol marketing is generally associated with alcohol use, including higher-risk drinking, among a diverse adult sample. It is important to caution, however, that the cross-sectional design means that our findings should not be taken as evidence that increased awareness of marketing causes increased drinking. Nevertheless, the broadly consistent relationship between awareness and alcohol use across marketing sources and activities suggests that there is some degree of relationship between them. Previous research has highlighted how alcohol marketing seeks to influence myriad psychological, behavioural, social, and societal mechanisms, which combine and interact to directly and indirectly shape and reinforce consumption among consumers.^11^^,^^64^^,^^65^ Research has also highlighted that some alcohol marketing may target heavier drinkers,^64^ a group that accounts for most alcohol sales revenue in the UK.^66^ It is therefore likely arguments that marketing is a contributory factor to increased consumption, and arguments that those consuming at higher levels have heightened exposure and receptivity to marketing messages that reinforce consumption, both have merit in explaining the impact of marketing, with the nature and direction of the effect varying by context, marketing activity, and population group.

### Limitations of this study

While a nonprobability online panel provided a cost- and time-effective means of recruiting a large sample, limitations around panel representativeness and self-selection or nonresponse may constrain generalizability to the wider UK population. The self-reported data may also be subject to recall errors or response biases, albeit our development stages sought to minimise this. Our marketing measures also only captured whether participants recalled any awareness, not how often exposure occurred. Measuring frequency of awareness was considered too onerous for participants and susceptible to moderation by extraneous variables we did not have the capacity to measure. We also only measured whether participants had seen marketing or price deals, but not whether they had engaged with these (e.g. purchased price deals) or their affective responses to marketing content. We also only report associations between awareness and alcohol use. While associations with other covariates are likely of interest, such as whether marketing awareness is correlated with age, reporting these was beyond the limits of this paper. Finally, our reporting is not exhaustive of all activities used to market alcohol, as our analysis excludes activities from the survey that were not measured in a past-month timeframe (e.g. ownership of branded merchandise) or where it could not be definitively known that the activity represented marketing (e.g. depictions of alcohol in TV shows/films or seeing influencers posting about alcohol on social media).

### Conclusion

Adults in the UK recall seeing alcohol marketing through a wide range of activities from both companies and brands and places that sell alcohol. Compared to lower-risk drinkers, awareness was generally lower among nondrinkers and higher among higher-risk drinkers.

## Supplementary Material

Supplementary_file_one_v2_fdaf118

## Data Availability

The data underlying this article may be shared on reasonable request to the corresponding author.

## Notes

**Nathan Critchlow,** Research Fellow

**Anne Marie MacKintosh,** Associate Professor

**Allison Ford,** Associate professor
