# Supplementary material for: Where do adults see alcohol marketing? Insight from a cross-sectional survey in the United Kingdom
Source: J Public Health (Oxf). 2025 Sep 26;47(4):e586–96. doi: 10.1093/pubmed/fdaf118 (PMC12670007; doi:10.1093/pubmed/fdaf118)
Supplement: Supplementary_file_one_v2_fdaf118 [file supplementary_file_one_v2_fdaf118.docx]

**Supplementary file one: Wording of marketing activities and special price offers, as presented to participants in the survey**

| **As reported in Table 2: advertising from companies and brands** | **As worded in the survey** |
| --- | --- |
| Posters or billboards (e.g. in the street or on public transport) | In the past month, have you seen adverts for alcohol on posters or billboards, such as those:   - In the street - By the roadside - On buses, taxis, trains, or trams - On bus/taxi shelters - At train/underground stations or tram stops |
| Broadcast TV (incl. programme sponsorship) | In the past month, have you seen adverts for alcohol on broadcast TV, including alcohol brands sponsoring TV programmes |
| Catch-up/streaming TV services | In the past month, have you seen adverts for alcohol on catch-up/streaming TV services |
| Popping up on online (e.g. browsing websites or using apps) | In the past month, have you seen adverts for alcohol popping up online (e.g. when browsing websites, using smartphone apps, or playing games on a smartphone) |
| Newspapers or magazines | In the past month, have you seen adverts for alcohol in newspapers or magazines |
| Podcasts/audio streaming (incl. show/episode sponsorship) | In the past month, have you heard adverts for alcohol on podcasts or music/audio streaming services, including alcohol brands sponsoring podcast shows/episodes |
| Radio | In the past month, have you heard adverts for alcohol on the radio |
| Cinema | In the past month, have you seen adverts for alcohol at the cinema |
| Routing and presentation notes: For all activities, except posters/billboards and adverts popping up online, participants were initially asked whether they had engaged with that form of media in the past month (e.g. watched broadcast TV channels, listened to the radio, or been to the cinema). Only those who had were subsequently asked about awareness of alcohol advertising through that activity. Those who had not engaged with that media activity are coded as ‘no’ for awareness. Each activity was presented on a separate survey page. | |

| **As reported in Table 2: wider marketing from companies and brands** | **As worded in the survey** |
| --- | --- |
| Brand names or logos on packaging | In the past month, have you seen alcohol brand names or logos on either…   - Bottles or cans - Outer packs (e.g. cardboard boxes or metal tubes) |
| Sports sponsorship (e.g. teams, events, tournaments) | In the past month, have you seen sports teams or sport events/tournaments which are sponsored by, or connected to, an alcohol brand. |
| Celebrity endorsement | In the past month, have you seen famous people, such as actors or music artists, endorsing or connected to an alcohol brand. |
| Cultural event sponsorship (e.g. concerts, festivals) | In the past month, have you seen events, such as concerts or festivals, which are sponsored by, or connected to, an alcohol brand |
| Competitions or giveaways linked to an alcohol company | In the past month, have you seen competitions or giveaways linked to an alcohol company or brand (e.g. opportunities to win free products, event tickets, or experiences) |
| Social media posts by alcohol companies or brands | Which, if any, of the following groups have you seen posting about alcohol on social media in the past month? Alcohol companies or brands |
| Free trials, tasters, or samples | In the past month, have you seen free trials, tasters, or samples of alcohol being offered. |
| Routing and presentation notes: Participants were initially asked how often they used social media. Only those who reported active social media use were asked about seeing alcohol companies and brands posting on social media. Those who did not report active social media use were coded as ‘no’ for awareness of social media posts from alcohol companies and brands. Most activities were presented on a separate survey page. The exception was social media posts by alcohol companies and brands, which was presented in a list alongside other sources of posting about alcohol on social media (e.g. friends, family, other people you know personally) | |

| **As reported in Table 3: Marketing from off-trade retailers (shops)** | **As worded in the survey** |
| --- | --- |
| Display in shops (e.g. end of aisles, at checkouts) | In the past month, have you seen displays of alcohol in shops, such as:   - At the end of aisles - At checkouts - As part of special or seasonal displays |
| Signs or posters (e.g. on shop windows, doors, or shelves) | In the past month, have you seen signs or posters in shops about alcohol, such as:   - Posters on shop windows/shop doors - Signs on shelves - Stickers on shop floors/fridge doors |
| Store leaflets, flyers, or magazines which show alcohol | In the past month, have you seen store leaflets, flyers, or magazines which show alcohol (e.g. [example title names from leading UK retailers]) |
| Social media posts about alcohol from shops | Which, if any, of the following groups have you seen posting about alcohol on social media in the past month? Shops (e.g. supermarkets) |
| Routing and presentation notes: Participants were initially asked how often they used social media. Only those who reported active social media use were asked about seeing shops posting about alcohol on social media. Those who did not report active social media use were coded as ‘no’ for awareness of social media posts about alcohol from shops. Most activities were presented on a separate survey page. The exception was social media posts about alcohol from shops, which was presented in a list alongside other sources of posting about alcohol on social media (e.g. friends, family, other people you know personally). | |

**Before answering questions about shops, participants were shown the following definition:**

Now thinking about things you may have seen inside and at shops in the past month.

By shops, we mean physical stores where people can buy alcohol to take away, such as:

- Supermarkets
- Convenience stores
- Newsagents
- Off-licences
- Petrol stations or garage forecourts

| **As reported in Table 3: Marketing from online retailers** | **As worded in the survey** |
| --- | --- |
| E-mail or app notifications from supermarkets or online retailers | Which, if any, of the following have you received in the past month? Please select all that apply: E-mail or app notifications about alcohol from a supermarket or online retailer |
| Leaflets or flyers about an online retailer/subscription service | Which, if any, of the following have you received in the past month? Please select all that apply: Leaflets or flyers about an online alcohol retailer/alcohol subscription service (e.g. in the packaging of items you have ordered) |
| Social media posts by specialist retailers/subscription services | Which, if any, of the following groups have you seen posting about alcohol on social media in the past month? Specialist alcohol retailers or subscription services |
| Routing and presentation notes: Participants were initially asked how often they used social media. Only those who reported active social media use were asked about seeing specialist alcohol retailers or subscription services posting on social media. Those who did not report active social media use were coded as ‘no’ for awareness of social media posts from specialist alcohol retailers or subscription services. Questions about e-mail/app notifications and leaflets or flyers were presented on the same survey page, and participants were asked to tick all that applied, or indicate ‘none of the above’ or ‘don’t know’. Posts on social media were presented in a list alongside other sources of posting about alcohol on social media (e.g. friends, family, other people you know personally) | |

**Before answering questions about online retailers, participants were shown the following definition:**

Now thinking about online retailers where people can order alcohol for delivery, such as:

- Supermarket websites/apps (e.g. [example of lead retailer] home delivery/click and collect)
- Alcohol retailers or subscription services (e.g. [example of leading UK brands])
- Takeaway or grocery delivery apps (e.g. [examples of leading UK brands])
- Other online retailers (e.g. [examples of leading UK brands])

| **As reported in Table 3: Marketing from on-trade venues (e.g. pub, bars, clubs)** | **As worded in the survey** |
| --- | --- |
| Brand names or logos *inside* a venue (e.g. drinks mats) | In the past month, have you seen alcohol brand names or logos inside a venue (e.g. on beer taps, drinks glasses, drinks mats, or bottles/cans behind the bar) |
| Brand names or logos *outside* a venue (e.g. signage) | In the past month, have you seen alcohol brand names or logos outside a venue (e.g. on barriers around outside seating areas or signage on a venue) |
| Posters, flyers, or leaflets that promote a venue | In the past month, have you seen posters, flyers, or leaflets which promote a venue that sells alcohol |
| Social media posts about alcohol from venues | Which, if any, of the following groups have you seen posting about alcohol on social media in the past month? Venues (e.g. pubs, bars, clubs, nightclubs). |
| Routing and presentation notes: Participants were initially asked how often they used social media. Only those who reported active social media use were asked about seeing social media posts from venues about alcohol in the past month. Those who did not report active social media use were coded as ‘no’ for awareness of venues posting about alcohol on social media. Most activities were presented on a separate survey page. The exception was social media posts, which was presented in a list alongside other sources of posting about alcohol on social media (e.g. friends, family, other people you know personally) | |

**Before answering questions about venues, participants were shown the following definition:**

Now thinking about venues where people can purchase alcohol to drink on the premises, such as:

- Pubs
- Bars
- Nightclubs
- Restaurants
- Social clubs

| **As reported in Table 4: Price deals from off-trade retailers (shops)** | **As worded in the survey** |
| --- | --- |
|  | Thinking about special price deals that shops might offer on alcohol. Which, if any, of the following have you seen in the past month? |
| Discounts linked to shop reward/membership schemes | Discounts linked to shop reward/membership schemes (e.g. [examples of loyalty schemes from leading UK retail brands]) |
| Multi-buy/bulk discounts (e.g. 'three bottles of beer for £5') | Multi-buy or bulk discounts (e.g. ‘three bottles of beer for £5’) |
| 'Dine-in' deals including alcohol (e.g. 'dinner for two for £10') | ‘Dine-in’ food and drink deals which include alcohol (e.g. ‘dinner for two for £10’) |
| Price reductions (e.g. '30% off' or ‘price-matching’) | Price reductions (e.g. ‘30% off’, ‘rollbacks’, price matching, or ‘reduced to clear’) |
| Routing and presentation notes: All special price deals for shops were presented in a list on the same survey page, and participants were asked to tick all that apply, or select ‘none of the above’ or ‘don’t know’. | |

| **As reported in Table 4: Price deals from online retailers** | **As worded in the survey** |
| --- | --- |
|  | Thinking about special price deals that online retailers might offer on alcohol. Which, if any, of the following have you seen in the past month? |
| Multi-buy/bulk discounts (e.g. ‘6 for £10 or 12 for £15’) | Multi-buy or bulk discounts (e.g. ‘6 cans for £10 or 12 cans for £15’) |
| Price reductions (e.g. 'save 10%' or 'was £13.50, now £11') | Price reductions (e.g. ‘save 10%’ or ‘was £13.50, now £11’) |
| Introductory subscription offers (e.g. ‘first box half price’) | Introductory offers for subscription services (e.g. ‘first box half price’) |
| Discounts for regular deliveries (e.g. 'subscribe and save') | Discounts for signing up to regular deliveries (e.g. ‘subscribe and save’) |
| Discount codes (e.g. 'enter Oct24 at checkout for £10 off’) | Discount codes (e.g. ‘enter Oct24 at checkout for £10 off’). |
| Routing and presentation notes: All special price deals for online retailers were presented in a list on the same survey page, and participants were asked to tick all that apply, or select ‘none of the above’ or ‘don’t know’. | |

| **As reported in Table 4: Price deals from on-trade venues (e.g. pub, bars, clubs)** | **As worded in the survey** |
| --- | --- |
|  | Thinking about special price deals that venues might offer on alcohol. Which, if any, of the following have you seen in the past month? |
| Food and drink combination deals (e.g. ‘beer and burger’) | Food and drink combination deals which include alcohol (e.g. ‘beer and burger for £10’) |
| Multi-buy/bulk discounts (e.g. ‘two-for-one drinks’) | Multi-buy or bulk discounts (e.g. ‘two-for-one drinks’ or sharing formats such as cocktail trees or beer pitchers/towers) |
| Temporary price reductions (e.g. ‘early bird’ offers) | Temporary price reductions (e.g. midweek discounts or ‘early bird’ offers) |
| Package deals which include alcohol | Package deals which include alcohol (e.g. nightclub packages which include entry, a table/booth, and selection of drinks) |
| Discount vouchers/offers for venues which include alcohol | Discounts vouchers/offers for venues which include alcohol (e.g. [examples of leading UK discount voucher brands]) |
| Routing and presentation notes: All special price deals for venues were presented in a list on the same survey page, and participants were asked to tick all that apply, or select ‘none of the above’ or ‘don’t know’. | |
